# Quantitative Acetylome Analysis of Differentially Modified Proteins in Virulence-Differentiated *Fusarium oxysporum* f. sp. *cucumerinum* Isolates during Cucumber Colonization

**DOI:** 10.3390/jof9090920

**Published:** 2023-09-13

**Authors:** Ying Zhou, Xiaohong Lu, Jianjun Hao, Shidong Li

**Affiliations:** 1 State Key Laboratory for Biology of Plant Diseases and Insect Pests, Institute of Plant Protection, Chinese Academy of Agricultural Sciences, Beijing 100193, China; 2 School of Food and Agriculture, University of Maine, Orono, ME 04469, USA

**Keywords:** *Cucumis sativus*, lysine acetylation, proteome, pathogenesis, virulence evolution

## Abstract

*Fusarium oxysporum* f. sp. *cucumerinum* (*Foc*) is a prominent pathogen that adversely affects cucumber (*Cucumis sativus*) production. In the pathogen’s parasitic lifestyle, the pathogenesis and virulence evolution may be regulated by lysine acetylation, as demonstrated in many living organisms. However, its specific function in *Foc* remains poorly understood. In this study, the acetylome profiles of a mild virulence strain (foc-3b) and its derived virulence-enhanced strain (Ra-4) were analyzed before and post-inoculation on cucumber plants. In total, 10,664 acetylation sites were identified corresponding to 3874 proteins, and 45 conserved acetylation motifs were detected. Through comparison of the acetylomes, numerous differentially lysine-acetylated proteins were enriched in energy metabolism and protein processing processes, indicating the critical role of lysine acetylation during the transition from the saprotrophic lifestyle to the parasitic lifestyle. Comparative acetylome analyses on the two virulence-differentiated strains revealed that several differentially lysine-acetylated proteins were involved in pathways of defense response and energy metabolism. Ra-4 showed enhanced energy metabolism compared to foc-3b. This indicates that robust metabolic activity is required to achieve high virulence and facilitating adaptive evolution. Additionally, faster host responses are supported by an ample energy supply enhancing virulence. Thus, lysine acetylation plays a crucial role in the pathogenesis and virulence evolution of *Foc*.

## 1. Introduction

The *Fusarium oxysporum* species complex includes widespread soil-borne and facultative parasites causing wilt or rot diseases in various plants. These diseases can result in significant annual crop losses globally. The complex is composed of 106 well-characterized formae speciales [1,2]. One particular member of the complex, *Fusarium oxysporum* f. sp. *cucumerinum* (*Foc*), is responsible for causing Fusarium wilt of cucumber (*Cucumis sativus*). *Foc* infections result in foliar drooping, necrotic marks on the stem base, and plant death. The pathogen poses a substantial threat to cucumber production [3,4].

Unfortunately, effective measures for controlling Fusarium wilt are currently limited. Planting resistant cultivars have exhibited promising results as an economical method for controlling cucumber Fusarium wilt [5]. However, the monoculture of resistant cultivars often results in significant selection pressure on the pathogen. This, in turn, can drive the evolution of novel virulence traits in the pathogen and even result in the breakdown of host plant resistance [6,7]. Previous studies have demonstrated that successive passage on the resistant cultivar can cause a mild strain of *Foc* foc-3b to evolve into a more virulent strain Ra-4 [8]. Comparative transcriptome analysis of these two virulence-differentiated *Foc* strains has revealed differential expression of specific genes encoding acetyltransferases and deacetylases [9]. These findings suggest that acetylation or deacetylation processes may influence the evolution of virulence. However, the specific biological functions of acetylation or deacetylation in the virulence evolution of *Foc* remain unclear.

Being a facultative parasite, *Foc* undergoes significant protein expression changes during the transition from a saprophytic lifestyle to a parasitic lifestyle. Many genes in *Foc* are differentially expressed after infecting cucumber roots, compared to growing in potato broth medium [9]. Notably, these differentially expressed genes include some acetyltransferase and deacetylase coding genes [9], indicating that lysine acetylation may be involved in the regulation of lifestyle transition. However, the specific profile of acetylation during this transition remains unknown.

Protein acetylation, especially lysine acetylation (Kac) is a prevalent reversible protein post-translational modification (PTM) in both eukaryotic and prokaryotic organisms [10,11]. Kac is essential for crucial biological processes, such as enzymatic activity, protein stability, metabolism regulation, and protein–protein interactions [12,13,14]. Several plant pathogens, including *F. graminearum*, *Botrytis cinerea*, *Aspergillus flavus*, and *Phytophthora sojae* have had their Kac profiles studied, revealing the involvement of acetylated proteins in pathogenesis and highlighting essential roles of Kac in the regulation of virulence [15,16,17,18]. However, the exploration of lysine acetylation in *F. oxysporum* has been limited to secreted proteins and the process of conidiation in *F. oxysporum* f. sp. *lycoperisici* [19,20]. The overall profile of lysine acetylation of *F. oxysporum* is required for a comprehensive understanding of pathogenesis and virulence evolution.

In this study, the proteome-wide lysine acetylomes of the *Foc* mild virulence strain foc-3b and the virulence-enhanced strain Ra-4 were generated for both the parasitic lifestyle and the saprophytic lifestyle, respectively. Comparative acetylome analyses of two strains and two lifestyles were conducted to identify acetylated proteins potentially contributing to the pathogenesis and virulence evolution of *Foc*. This research provides a comprehensive profile of the *Foc* acetylome and elucidates the roles of Kac in the pathogenesis and virulence evolution in *Foc*. Thus, the objectives of this work were to understand the pathogenesis and virulence evolution of *Foc* in infecting cucumbers by analyzing acetylome profiles and corresponding metabolisms.

## 2. Materials and Methods

### 2.1. Plant Materials, Fungal Strains, and Interactions

The Zhongnong No. 6 (ZN6) cucumber variety, susceptible to *Foc*, was provided by the China Vegetable Seed Technology Co., Ltd. (Beijing, China, 2020). Cucumber seeds were soaked in 1% (*w*/*v*) NaClO solution for 5 min before being thoroughly rinsed with distilled water. Subsequently, the treated seeds were planted in square culture plates containing Murashige and Skoog (MS) medium [21] and cultivated in a greenhouse at 26 °C under a 16 h light/8 h dark cycle for 10 days. The *Foc* mild strain foc-3b (obtained from the Agricultural Culture Collection of China, strain ACCC39326) and the virulence-enhanced strain Ra-4 (previously studied in the Biocontrol of Soilborne Diseases Laboratory, Institute of Plant Protection, Chinese Academy of Agricultural Sciences) were utilized in this study [8]. The *Foc* strains were initially cultured on potato dextrose agar (PDA) plates at 26 °C for 5 days in the dark. Two mycelial plugs (5 mm in diameter) were cut from the edge of actively growing colonies of each strain and transferred to a separate flask containing 100 mL potato dextrose broth (PDB). Triplicate flasks were prepared for each strain at 26 °C in the dark with shaking at 180 rpm for 3 days.

After incubation, a portion of the culture was centrifuged at 8000× *g* for 20 min at 25 °C. The precipitate containing mycelia and spores was collected as samples and designated as W_0h and I_0h for the wild-type strain foc-3b and the induced virulence-enhanced strain Ra-4, respectively. These samples were stored at −80 °C. Each sample weighed two grams and three replicates were employed. The remaining cultures were filtered using a 25 μm pore size mesh to obtain spores for cucumber seedling inoculation. The 10-day-old cucumber seedlings were inoculated using the root-cutting method. Specifically, the root tips were cut off, and the wounded roots were soaked in 10^6^ spores/mL suspensions of *Foc* for 30 min. Subsequently, the seedlings were cultured at 26 °C in darkness. Six hours post-inoculation, the roots were collected and immediately stored at −80 °C. The root samples were designated as W_6h and I_6h, representing the root inoculated with the strains foc-3b and Ra-4, respectively. Each sample weighed three grams and three replicates were employed.

### 2.2. Protein Extraction, Digestion, and Peptides Analysis

To extract protein, the samples were grouped and ground. Protein extraction and trypsin digestion were conducted following a previously described protocol [22]. The extracted protein of each sample was 4.5 mg and digested by trypsin. Peptides were desalted with a Strata X C18 SPE column (Phenomenex, Torrance, CA, USA), vacuum freeze-dried, and re-dissolved in 0.5 M TEAB. Then, a TMT 6-plex Labelling Kit (Thermo Fisher Scientific, Rockford, IL, USA) was used to properly label the peptide samples. The tagged peptides were divided using high-performance liquid chromatography (HPLC) on an Agilent 300 Extend C18 (Agilent, Santa Clara, CA, USA) column (5 mm particles, 4.6 mm ID, 250 mm length). Using a linear gradient of 8% to 32% acetonitrile, the peptides were divided into 60 segments over the course of 60 min, with one-minute intervals. The peptides were divided into 12 or more portions and dehydrated using a vacuum freeze centrifuge (Eppendorf, Hamburg, Germany). The tagged peptides of each sample were three micrograms.

To concentrate lysine acetylation peptides, the tagged peptides were dematerialized in IP buffer (100 mM NaCl, 1 mM EDTA, 50 mM Tris-HCl, 0.5% NP-40, pH 8.0), and gently shaken for 10 h at 4 °C with pre-treated PTM104 anti-acetyl lysine antibody resins (PTM Bio, Hangzhou, China). The resins were treated with IP buffer four times, ddH_2_O twice, then washed with 0.1% trifluoroacetic acid thrice to elute binding peptides. Following the instructions supplied by Millipore’s C18 Zip Tips (Shanghai, China), the combined fractions were vacuum freeze-dried and desalted.

The Kac-enriched peptides were dissolved and separated on an EASY-nLC 1000 UPLC System (Agilent, Santa Clara, CA, USA) following the protocol formerly described by Jiang et al. [22]. Then, the peptides were subjected to nanospray ionization (NSI) and analyzed with a Q Exactive^TM^ HF-X apparatus (Thermo Scientific, San Jose, CA, USA) that was linked with the UPLC. The MS1 spectra were acquired adopting an orbitrap with a mass resolution of 120,000 and an *m*/*z* scan range of 350–1400. The *m*/*z* scan range of MS2 spectra is capped at 100, while the mass resolution is 30,000. One MS scan was accompanied by 20 MS/MS scans in a data-dependent procedure for the upper 20 precursor ions over the threshold ion count of 100,000 in the MS measurement scanning with 15 s dynamic exclusion, and 28% high-energy C-trap dissociation in the electrospray operated at 2200 volts. The ion trap was protected against being overfilled by setting the automatic gain control to 100,000 and imposing a maximum injection time of 100 milliseconds.

Three independent experiments were performed. To minimize bias caused by protein abundance, quantitative proteome normalization was implemented. A *t*-test was employed to assess the significance of the fold-change in the differential modification ratio of acetylated sites across treatments. Proteins with a fold-change above 1.5 and a significance level below 0.05 were identified as up-regulated, whereas those with a fold-change below 1/1.5 were identified as down-regulated. Principal component analysis, Pearson’s correlation coefficient, and relative standard deviation were employed to analyze the data from the three repeated experiments to assess the impact on quantitative repeatability.

### 2.3. Bioinformatics Analyses

To locate matches in the generated MS/MS data, MaxQuant (v1.6.15.0) was utilized to browse the NCBI and UniProt *F. oxysporum* (246,924 sequences) databases, in addition to a reverse decoy database. Trypsin/P was specified as the cleavage enzyme, up to four missed cleavages were permitted. Peptides were set with a minimum of seven amino acids in length, along with a maximum of five modifications per peptide. The mass error of the precursor ions was set at 20 ppm in the initial scan, five ppm in the main scan, and for the fragment ions it was 0.02 Da. Cys carbamidomethylation was chosen as the fixed modification, whereas Met oxidation, Lys acetylation, and protein N-terminal acetylation were chosen as variable modifications. TMT 6-plex was chosen for quantitative analysis. Limits on the false discovery rate (FDR) for peptides, proteins, and modification sites were all set to 1%, and the site localization probability was adjusted to be higher than 0.75. For motifs analysis, we used the motif-x algorithm in MoMo (https://meme-suite.org/meme/tools/momo (accessed on 15 December 2020)), which requires the presence of 10 amino acids upstream and downstream of the acetylated region in all protein sequences. The STRING database (version 10.5) was queried to obtain information on physical and functional protein–protein interactions involving all identified acetylated proteins. Only interactions with confidence values greater than 0.7 were considered.

The analysis of lysine-acetylated peptides and proteins was performed using various bioinformatics tools. The identified proteins were categorized using UniProt-GOA (http://www.ebi.ac.uk/GOA/ (accessed on 15 December 2020)) and were further annotated using functional descriptions of Gene Ontology domains through InterProScan (http://www.ebi.ac.uk/interpro/ (accessed on 15 December 2020)) for proteins lacking annotation in UniProt-GOA. The Kac proteins were separated into molecular function, cellular component, and biological process classes based on GO annotation (http://www.geneontology.org/ (accessed on 15 December 2020)). Protein localization prediction was the reason for the creation of WoLFPSORT (https://wolfpsort.hgc.jp/ (accessed on 15 December 2020)). The functional pathways of lysine-acetylated proteins were annotated using the Kyoto Encyclopedia of Genes and Genomes (KEGG) database (https://www.genome.jp/kegg/ (accessed on 15 December 2020)). In contrast, the Eukaryotic Orthologous Group (KOG) database (https://mycocosm.jgi.doe.gov/help/kogbrowser.jsf (accessed on 15 December 2020)) was employed to map acetylated proteins.

The background database settings were configured to include protein sequences from all databases, while other parameters were retained at their default settings. To assess the percentage of differently lysine-acetylated proteins in individual protein annotation groups, Fisher’s two-tailed exact test was applied. Enrichment analysis was conducted, and annotation categories with Fisher’s exact test *p*-values lower than 0.05 were considered to be significantly enriched.

To identify the differentially lysine-acetylated sites and proteins in *Foc*, the parasitic lifestyle (cucumber seedlings inoculated, samples W_6h and I_6h) was compared with the saprophytic growth of related *Foc* strains (foc-3b and Ra-4 grown in PDB, designated as W_0h and I_0h). Additionally, the induced virulence-enhanced strain Ra-4 (samples I_0h and I_6h) was compared to the wild-type strain foc-3b (samples W_0h and W_6h) of the related lifestyle. Samples were compared as W_6h versus W_0h, I_6h versus I_0h, I_0h versus W_0h, and I_6h versus W_6h.

## 3. Results

### 3.1. Comprehensive Analysis of Identified Lysine-Acetylated Proteins in Foc

**Figure 1:**
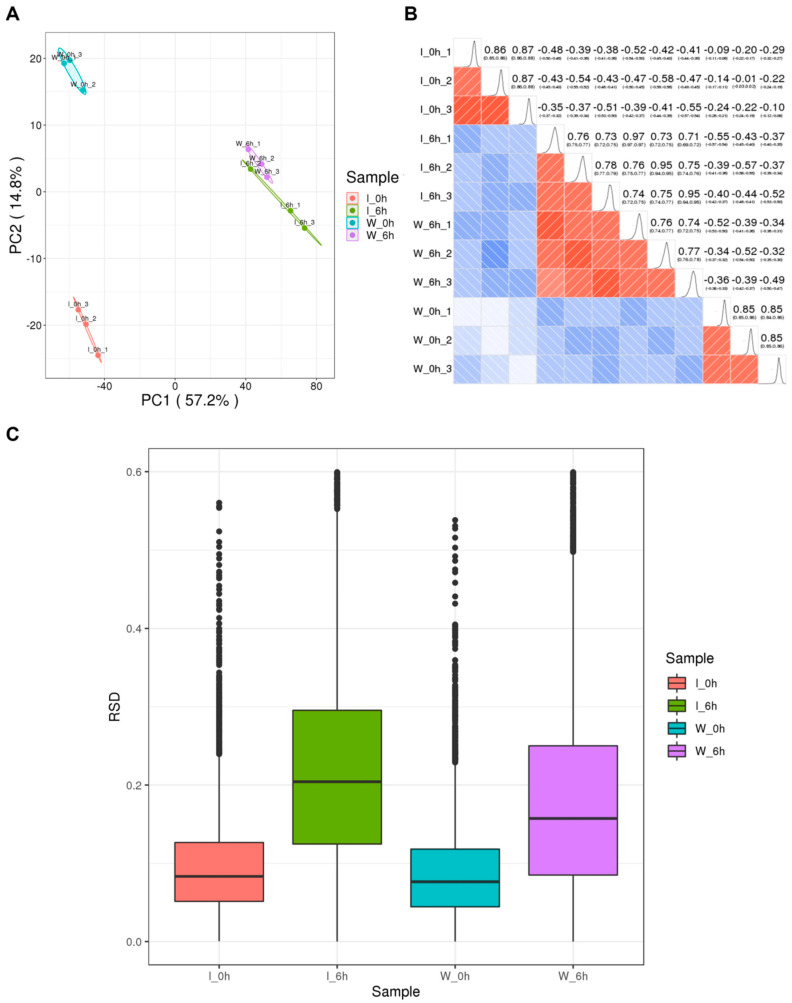
Repeatability tests of *Fusarium oxysporum* f. sp. *cucumerinum* samples: (**A**) Principal component analysis; (**B**) heatmap of Pearson correlation; (**C**) distribution of relative standard deviation. The samples were from *F. oxysporum* f. sp. *cucumerinum* strains foc-3b (W) and Ra-4 (I) during the saprophytic lifestyle in potato dextrose broth cultures (0 h) or parasitic lifestyle at 6 h post-inoculation (6 h) on *Cucumis sativus* roots.

**Figure 2:**
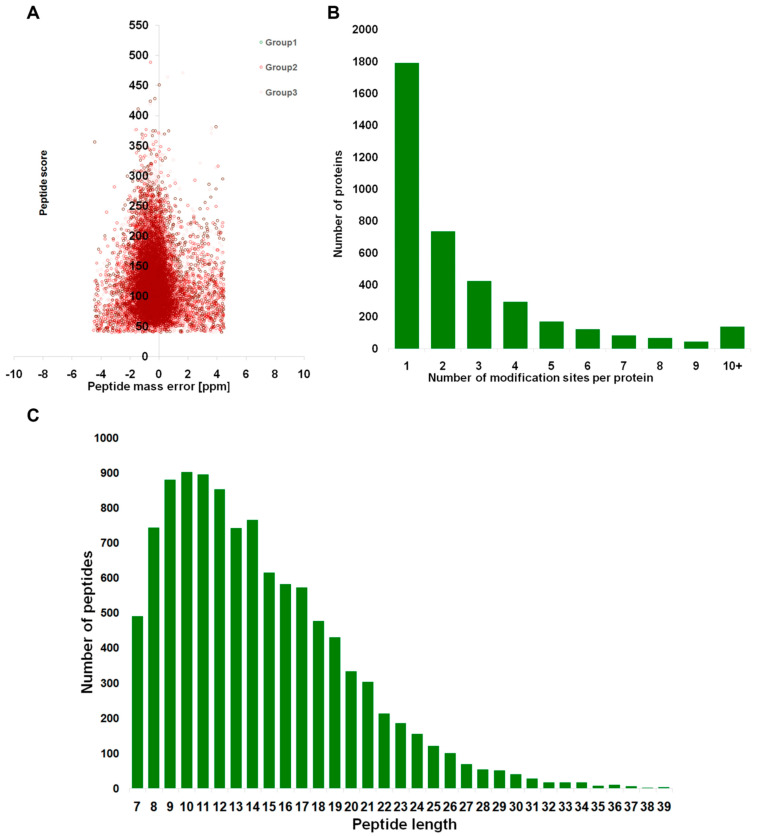
Comprehensive analysis of all identified lysine-acetylated peptides in *Fusarium oxysporum* f. sp. *cucumerinum* strains foc-3b and Ra-4: (**A**) Mass error distribution; (**B**) proteins modifications distribution; (**C**) peptide length (amino acids) distribution.

The quantitative Kac data were collected during the parasitic and saprophytic lifestyles of foc-3b and Ra-4 strains, which were consistent in repeatability tests (Figure 1). Most mass errors were below 5 ppm (Supplementary Table S1, Figure 2A), indicating accurate MS analysis of the data. A total of 10,664 Kac sites were identified from 3874 different proteins (Supplementary Table S1), accounting for 13% of all proteins in *Foc*. Nearly half of Kac proteins (46.3%) contained one acetylated site, with an average of 2.7 acetylated sites per protein (Figure 2B). The length distribution of tryptic peptides in protein samples indicated that most of the peptides ranged from 7 to 20 amino acids in length (Figure 2C).

### 3.2. Analysis of Lysine-Acetylated Motifs

**Figure 3:**
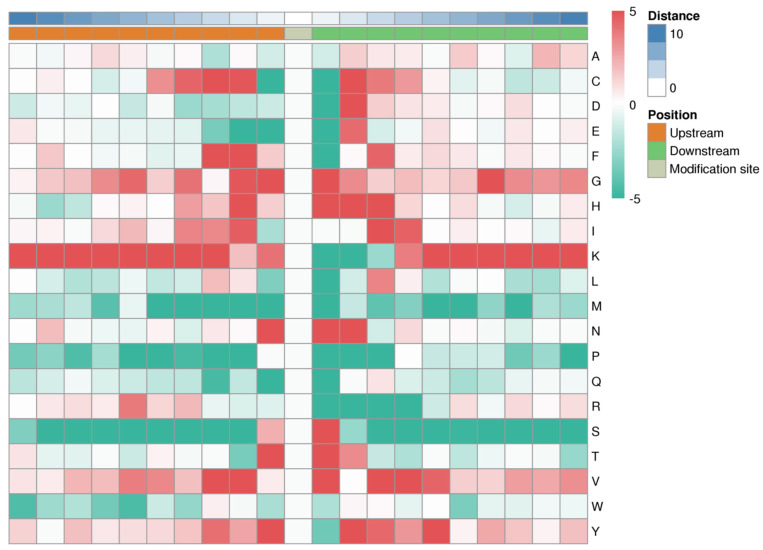
Motif-X intensity map of all identified lysine-acetylated sites in *Fusarium oxysporum* f. sp. *cucumerinum* strains foc-3b and Ra-4. On the right side, the interpretation of reference colors for frequency (from red to green), distance, and position.

Motif-X intensity map (Figure 3) evaluated amino acid sequences flanking lysine-acetylated sites: glycine (G), histidine (H), lysine (K), valine (V), and tyrosine (Y) residues had high frequencies, whereas methionine (M), proline (P), and serine (S) residues had low frequencies. In addition, 45 conserved motifs were uncovered, and the motifs KacAD, TKacL, and KacPD were more specific based on the motif score (Supplementary Table S2).

### 3.3. Characteristics of Differentially Lysine-Acetylated Sites and Proteins in Foc

**Figure 4:**
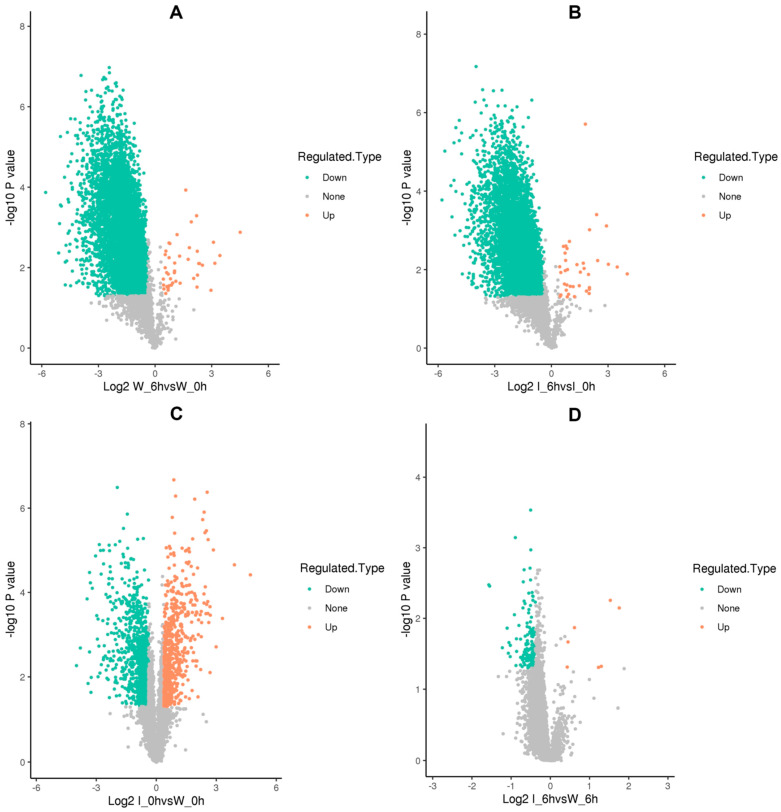
Volcano plot of differentially lysine-acetylated sites in *Fusarium oxysporum* f. sp. *cucumerinum* under different comparisons: (**A**) W_6h versus W_0h; (**B**) I_6h versus I_0h; (**C**) I_0h versus W_0h; (**D**) I_6h versus W_6h. Interpretation of the reference colors: orange = up-regulated, green = down-regulated, grey = unchanged. W_0h: wild-type strain foc-3b before inoculation. I_0h: Virulence-induced strain Ra-4 before inoculation. W_6h: wild-type strain foc-3b at 6 h post-inoculation. I_6h: Virulence-induced strain Ra-4 6 h post-inoculation.

At the early stage of the parasitic lifestyle (6 h post-inoculation) of the *Foc* mild strain foc-3b (W_6h), 43 Kac sites of 43 proteins were found to be up-regulated, while 4999 Kac sites of 2076 proteins were down-regulated, compared to the saprotrophic lifestyle (W_0h) (Figure 4A, Supplementary Table S3). A volcano plot was prepared to illustrate protein differences after a log2 transformation on the horizontal axis, and a log10 transformation of p-values represents the significance of various tests on the vertical axis.

For the virulence-enhanced strain Ra-4, 38 Kac sites on 37 proteins were up-regulated. In comparison, 4819 Kac sites on 2029 proteins were down-regulated during the early stage of the parasitic lifestyle (I_6h) compared to the saprotrophic lifestyle (I_0h) (Figure 4B, Supplementary Table S3). When compared to the mild strain foc-3b, in the saprotrophic lifestyle (I_0h versus W_0h), the virulence-enhanced strain Ra-4 exhibited up-regulation of 624 Kac sites across 351 proteins and down-regulation of 1175 Kac sites across 597 proteins (Figure 4C, Supplementary Table S3). However, in the parasitic lifestyle (I_6h versus W_6h), Ra-4 exhibited only 7 up-regulated Kac sites on 7 proteins and 134 down-regulated Kac sites on 126 proteins (Figure 4D, Supplementary Table S3).

The comparisons of W_6h versus W_0h and I_6h versus I_0h indicated that the number of acetylated proteins with down-regulated Kac sites exceeded the number of acetylated proteins with up-regulated Kac sites, suggesting that a significant portion of acetylated proteins may carry out their functions through deacetylation processes during the parasitic lifestyle in *Foc*.

### 3.4. Functional Categorization and Subcellular Location of Differentially Lysine-Acetylated Proteins in Foc

**Figure 5:**
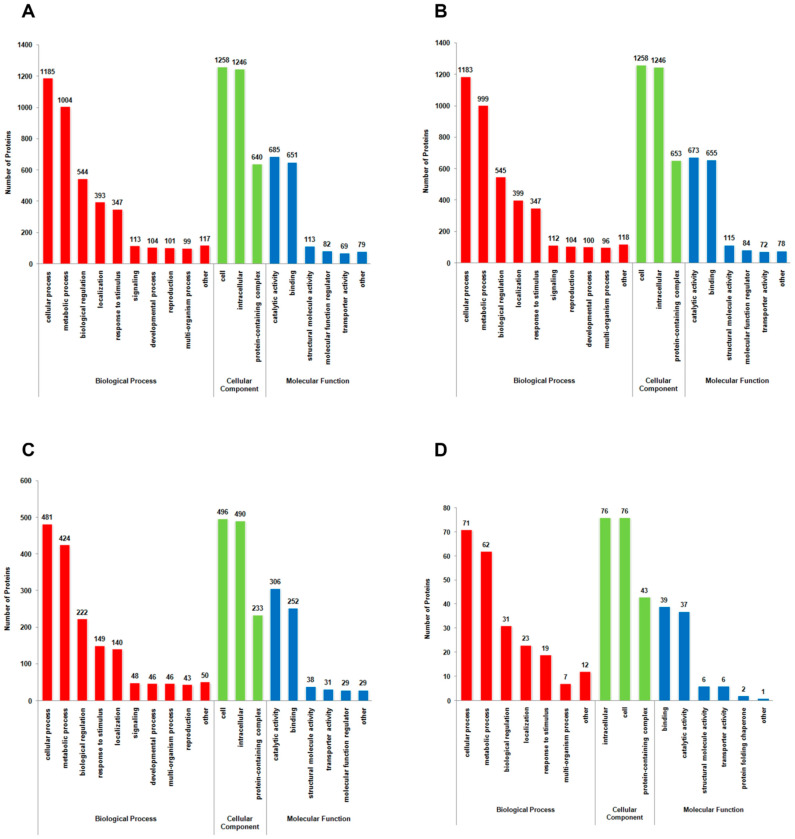
*Fusarium oxysporum* f. sp. *cucumerinum* Gene Ontology (GO) functional description of differentially lysine-acetylated proteins across various comparisons: (**A**) W_6h versus W_0h; (**B**) I_6h versus I_0h; (**C**) I_0h versus W_0h; (**D**) I_6h versus W_6h. Numbers above the bar charts indicate the lysine-acetylated proteins. W_0h: wild-type strain foc-3b before inoculation. I_0h: Virulence-induced strain Ra-4 before inoculation. W_6h: wild-type strain foc-3b at 6 h post-inoculation. I_6h: Virulence-induced strain Ra-4 at 6 h post-inoculation.

The mild strain foc-3b and the virulence-enhanced strain Ra-4 possessed similar GO and KOG functional categorization of differentially acetylated proteins between the parasitic and saprotrophic lifestyles (Figure 5A,B, Supplementary Table S4). The differentially acetylated proteins were assigned to 8830 GO terms for the mild strain foc-3b and 8837 GO terms for the virulence-enhanced strain Ra-4. Among these terms, the top two were cellular components ‘cell’ and ‘intracellular’, followed by the biological processes ‘cellular process’ and ‘metabolic process’. These four terms accounted for over 50% of all the assigned GO terms. Additionally, these differentially acetylated proteins were assigned to 1745 KOG terms for foc-3b and 1709 KOG terms for Ra-4. The dominant terms were ‘posttranslational modification, protein turnover, and chaperones’ and ‘translation, ribosomal structure, and biogenesis’ in the category of ‘cellular process and signaling’ (Table 1, Supplementary Table S6). Subcellular localization analysis revealed that 77% of the differentially acetylated proteins were located in the cytoplasm, nucleus, and mitochondria (Table 2, Supplementary Table S5).

Comparative analysis between the mild strain foc-3b and its virulence-enhanced strain Ra-4 indicated that a higher number of differentially acetylated proteins were present during the saprotrophic lifestyle than during the parasitic lifestyle (Figure 5C,D, Supplementary Table S4). In the saprotrophic lifestyle, 3553 GO terms and 750 KOG terms were assigned, while only 511 GO terms and 118 KOG terms were assigned in the parasitic lifestyle. The top four GO terms were also ‘cell’, ‘intracellular’, ‘cellular process’, and ‘metabolic process’, which accounted for over 50% of all the assigned GO terms. However, ‘amino acid transport and metabolism’ and ‘energy production and conversion’ were the top two KOG terms assigned for differentially acetylated proteins during the saprotrophic lifestyle, which differed from the top two KOG terms in other comparisons (Table 1, Supplementary Table S6). Subcellular localization analysis showed that most of the differentially acetylated proteins were located in the cytoplasm, nucleus, and mitochondria (Table 2, Supplementary Table S5).

### 3.5. Analysis of Functional Enrichment of Differentially Lysine-Acetylated Proteins in Foc

**Figure 6:**
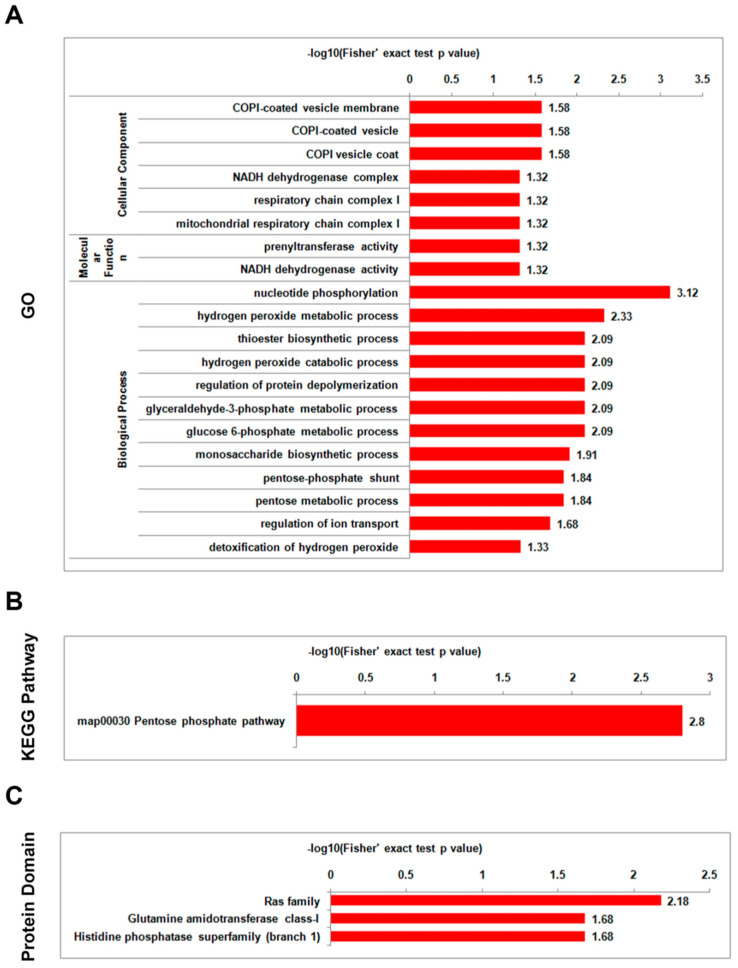
Enrichment analysis of differentially lysine-acetylated proteins in *Fusarium oxysporum* f. sp. *cucumerinum* for the comparison between 6 h post-inoculation (W_6h) and pre-inoculation (W_0h) in the wild-type strain foc-3b: (**A**) Gene Ontology (GO); (**B**) Kyoto Encyclopedia of Genes and Genomes (KEGG) pathway; (**C**) protein domain.

**Figure 7:**
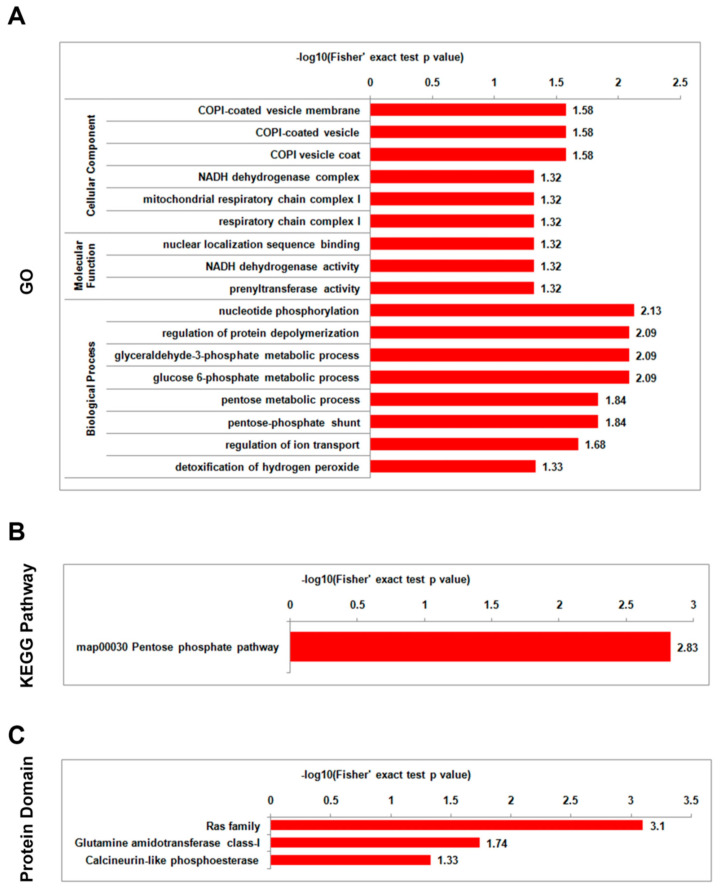
Enrichment analysis of differentially lysine-acetylated proteins in *Fusarium oxysporum* f. sp. *cucumerinum* for the comparison between 6 h post-inoculation (I_6h) and pre-inoculation (I_0h) in the virulence-enhanced strain Ra-4: (**A**) Gene Ontology (GO); (**B**) Kyoto Encyclopedia of Genes and Genomes (KEGG) pathway; (**C**) protein domain.

To determine the enrichment tendencies of differentially lysine-acetylated proteins, GO enrichment, KEGG pathway enrichment, and protein domain enrichment analyses were performed. Both the mild strain foc-3b and the virulence-enhanced strain Ra-4 had a similar functional enrichment categorization in GO, KEGG pathway, and protein domain analyses when comparing the parasitic lifestyle to the saprotrophic lifestyle. The differentially acetylated proteins were found to be significantly enriched in categories associated with energy metabolism and protein processing (Figure 6 and Figure 7, Supplementary Tables S7–S9). In terms of GO enrichment, the most enriched biological process for the differentially acetylated proteins was ‘nucleotide phosphorylation’, followed by ‘regulation of protein depolymerization’, ‘glyceraldehyde-3-phosphate metabolic process’, and ‘glucose 6-phosphate metabolic process’. Within the KEGG pathway enrichment analysis, there was a single term, ‘pentose phosphate pathway (PPP)’ that was significantly enriched. ‘Ras family’ was the top term in protein domain enrichment. Thus, the enrichment analysis of differentially lysine-acetylated proteins indicated their involvement in critical biological processes related to energy metabolism and protein processing during the transition from the saprotrophic lifestyle to the parasitic lifestyle in both the mild strain foc-3b and the virulence-enhanced strain Ra-4.

Additionally, in the GO enrichment analysis of mild strain foc-3b, the differentially acetylated proteins were also strongly enriched in four biological process terms ‘hydrogen peroxide metabolic process’, ‘thioester biosynthetic process’, ‘hydrogen peroxide catabolic process’, and ‘monosaccharide biosynthetic process’. These terms were not enriched in the virulence-enhanced strain Ra-4. In the GO enrichment analysis of virulence-enhanced strain Ra-4, the molecular function term ‘nuclear localization sequence binding’ was also absent in the mild strain foc-3b. In the protein domain enrichment analysis, the differentially acetylated proteins in the mild strain foc-3b were enriched in the term ‘histidine phosphatase superfamily (branch 1)’. In contrast, in the virulence-enhanced strain Ra-4, the term ‘calcineurin-like phosphoesterase’ was enriched. These findings indicate distinct functional characteristics associated with lysine-acetylated proteins between the mild strain foc-3b and the virulence-enhanced strain Ra-4.

**Figure 8:**
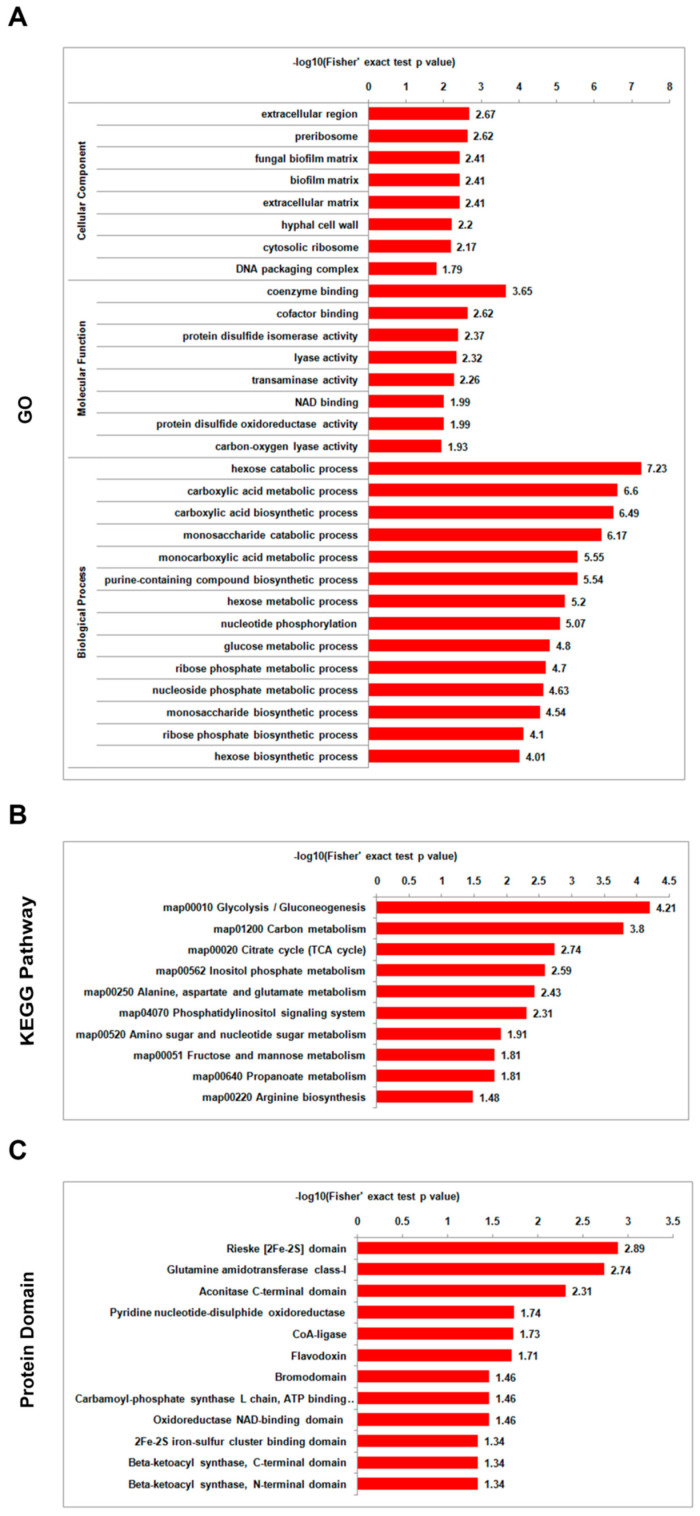
Enrichment analysis of differentially lysine-acetylated proteins in *Fusarium oxysporum* f. sp. *cucumerinum* for the comparison of the virulence-enhanced strain Ra-4 (I_0h) versus the wild-type strain foc-3b (W_0h) before inoculation: (**A**) Gene Ontology (GO); (**B**) Kyoto Encyclopedia of Genes and Genomes (KEGG) pathway; (**C**) protein domain.

During the saprotrophic lifestyle, comparative analysis between the mild strain foc-3b and its virulence-enhanced strain Ra-4 demonstrated that the differentially acetylated proteins were significantly enriched in categories associated with energy metabolism. In the GO enrichment analysis, the differentially acetylated proteins related to the biological process ‘hexose catabolic process’ were most enriched. These acetylated proteins were also enriched in cellular component terms such as ‘fungal biofilm matrix’ and ‘extracellular matrix’, suggesting their potential involvement in fungal resistance. In KEGG pathway enrichment analysis, the differentially acetylated proteins were most enriched in the term ‘glycolysis/gluconeogenesis’, which is a critical pathway involved in energy production. Furthermore, in protein domain enrichment analysis, the most enriched term was ‘Rieske (2Fe-2S) domain’, which is often associated with electron transfer in metabolic pathways (Figure 8, Supplementary Tables S7–S9). These findings highlight the importance of energy metabolism and related processes in the saprotrophic lifestyle of *Foc* strains.

**Figure 9:**
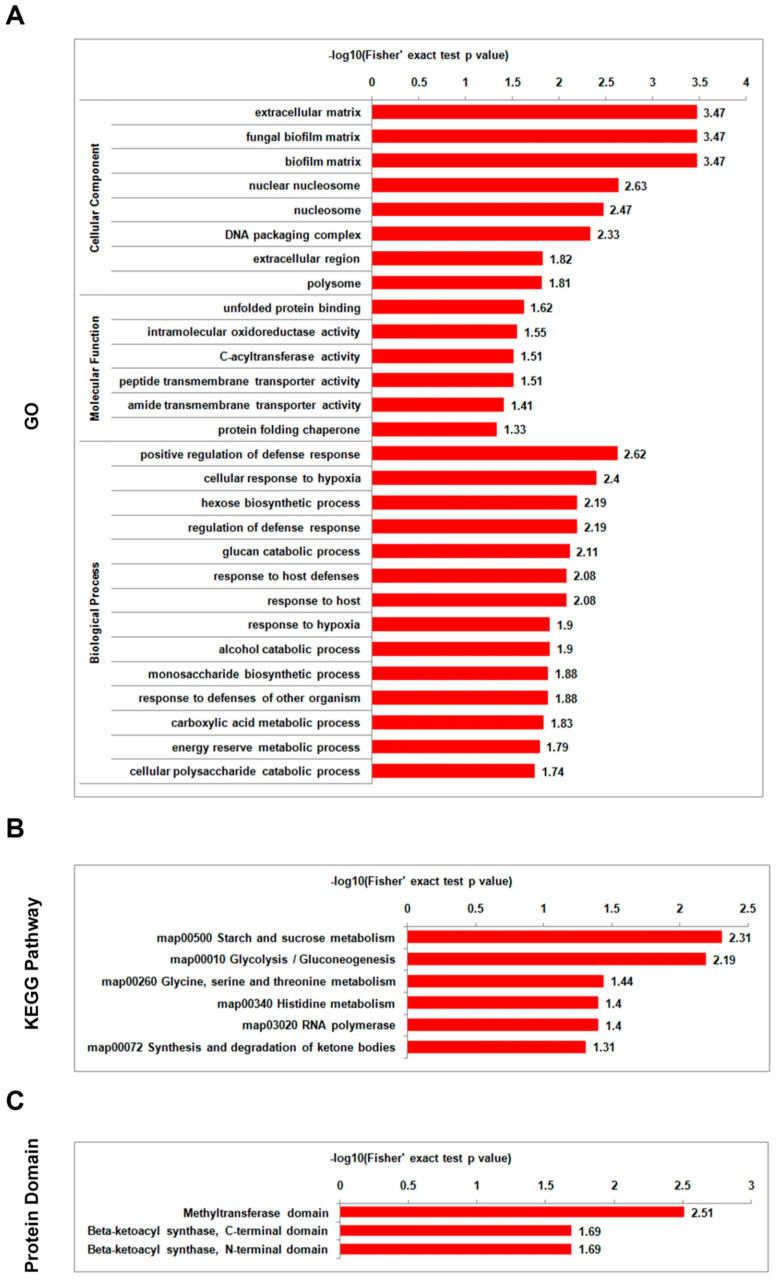
Enrichment analysis of differentially lysine-acetylated proteins in *Fusarium oxysporum* f. sp. *cucumerinum* for the comparison of the virulence-enhanced strain Ra-4 (I_6h) versus the wild-type strain foc-3b (W_6h) 6 h post-inoculation: (**A**) Gene Ontology (GO); (**B**) Kyoto Encyclopedia of Genes and Genomes (KEGG) pathway; (**C**) protein domain.

Comparative analysis between the mild strain foc-3b and its virulence-enhanced strain Ra-4 demonstrated that the differentially acetylated proteins were significantly enriched in the categories associated with defense response and energy metabolism during the parasitic lifestyle. In the GO enrichment analysis, the differentially acetylated proteins were primarily enriched in cellular component terms such as ‘extracellular matrix’, ‘fungal biofilm matrix’, and ‘biofilm matrix’. Additionally, the cellular component term ‘nuclear nucleosome’ and biological process term ‘positive regulation of defense response’ showed significant enrichment. Furthermore, the differentially acetylated proteins were also enriched in cellular component terms ‘nucleosome’, ‘DNA packaging complex’, and biological process terms ‘cellular response to hypoxia’, ‘hexose biosynthetic process’, ‘regulation of defense response’, and ‘glucan catabolic process’. In the KEGG pathway enrichment analysis, the differentially acetylated proteins were strongly associated with the terms such as ‘starch and sucrose metabolism’ and ‘glycolysis/gluconeogenesis’. The top term in the protein domain enrichment analysis was ‘methyltransferase domain’ (Figure 9, Supplementary Tables S7–S9). These findings indicate the involvement of differentially acetylated proteins in defense response and energy metabolism during the parasitic lifestyle of *Foc* strains.

### 3.6. Analysis of Protein Interaction Network of Differentially Lysine-Acetylated Proteins in Foc

To investigate the biological processes involved with differentially regulated acetylated proteins, networks of protein interactions were established.

**Figure 10:**
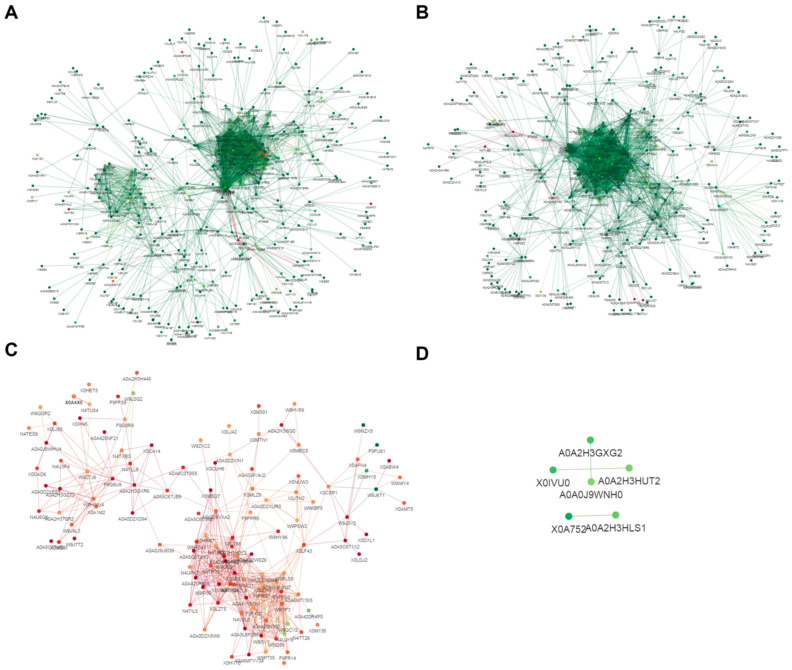
Protein–protein interaction network of differentially lysine-acetylated modification sites in *Fusarium oxysporum* f. sp. *cucumerinum* for the comparison: (**A**) W_6h versus W_0h; (**B**) I_6h versus I_0h; (**C**) I_0h versus W_0h; (**D**) I_6h versus W_6h. Interpretation of the reference colors: red = up-regulated, green = down-regulated. W_0h: wild-type strain foc-3b before inoculation. I_0h: Virulence-induced strain Ra-4 before inoculation. W_6h: wild-type strain foc-3b at 6 h post-inoculation. I_6h: Virulence-induced strain Ra-4 at 6 h post-inoculation.

For the *Foc* mild strain foc-3b (W_6h versus W_0h), the interaction network was comprised of 334 acetylated proteins, including 6 proteins with up-regulated Kac sites and 328 proteins with down-regulated Kac sites. Additionally, 210 acetylated proteins were found to have multiple interacting protein partners (Figure 10A, Supplementary Table S10). In the case of the virulence-enhanced strain Ra-4 (I_6h versus I_0h), the interaction network consisted of 291 acetylated proteins, with 5 proteins showing up-regulated Kac sites, 286 proteins showing down-regulated Kac sites, and 189 acetylated proteins having multiple interacted protein partners (Figure 10B, Supplementary Table S10). These protein interaction networks provide insights into the complex interplay and potential functional associations among differentially acetylated proteins in *Foc* strains during the transition from the saprotrophic lifestyle to the parasitic lifestyle.

Comparative analysis between the mild strain foc-3b and the virulence-enhanced strain Ra-4, during the saprotrophic lifestyle (I_0h versus W_0h), identified 117 acetylated proteins in the interaction network. This network included 109 proteins with up-regulated Kac sites and 8 proteins with down-regulated Kac sites. Among them, 93 acetylated proteins had multiple interacting protein partners (Figure 10C, Supplementary Table S10). However, during the parasitic lifestyle (I_6h versus W_6h), only 6 acetylated proteins with down-regulated Kac sites were identified in the interaction network, and all of them had a single protein partner (Figure 10D, Supplementary Table S10).

## 4. Discussion

In this pioneering study, we conducted comprehensive acetylome analyses based on the entire proteome of *Foc*, encompassing both parasitic and saprophytic lifestyles. We identified a remarkable 10,664 Kac sites and 45 highly conserved Kac motifs across 3874 distinct proteins of *Foc*, accounting for approximately 13% of all proteins. The large number of Kac sites, acetylated proteins, and the high-level acetylation ratio of *Foc* indicated the potential significance of Kac modification in the parasitic lifestyle and provided abundant acetylome data for further research. Notably, the number of Kac sites, acetylated proteins, acetylation ratio, and Kac motifs in *Foc* was much greater than formerly reported in other pathogenic fungi such as *F. graminearum* (577 Kac sites from 364 proteins, 2.73% acetylation ratio, 6 motifs) [15], *B. cinerea* (1582 Kac sites from 954 proteins, 5.82% acetylation ratio, 6 motifs) [16], and *Beauveria bassiana* (464 Kac sites from 283 proteins, 2.73% acetylation ratio, 10 motifs) [23]. The abundant Kac information in *Foc* can likely be linked to the inclusion of both the parasitic and saprophytic lifestyles in the present study, providing a comprehensive understanding of lysine acetylation in this pathogen.

Among the 45 highly conserved Kac motifs identified in the present study, some motifs are conserved among species, while others are specific to *Foc*. The two most prevalent motifs in *Foc* are the KacS motif, which has been reported in *Trichinella sprialis* [24], *A. flavus* [17], *Clonostachys chloroleuca* [22], and *F. oxysporum* f. sp. *lycoperisici* [19]; and the KacT motif, which has been reported in *T. sprialis* [24] and *C. chloroleuca* [22]. This suggests a high level of conservation of KacS and KacT motifs across these species. Interestingly, the top three most specific motifs identified in *Foc*, including TKacL, KacAD, and KacPD, have not been reported in other species. These specific motifs likely play crucial roles in the recognition of acetylation events in *Foc*, and proteins including these motif sites may be strictly regulated by acetylation and should be explored further. Lysine (K) was subjected to strict regulation through acetylation. Further exploration is required to uncover the functions and regulatory mechanisms associated with these motifs. We also observed that lysine residues were conserved in the vicinity of acetylated sites, which has also been found in *C. chloroleuca* [22]. However, the specific function of these conserved lysine residues throughout multiple positions flanking the Kac sites in *Foc* was not well known and required further investigation.

Comparative acetylome analyses of parasitic and saprophytic lifestyles of *Foc* revealed numerous differentially acetylated proteins involved in several essential biological processes, such as the pentose phosphate pathway (PPP), respiratory chain, protein processing, nucleotide phosphorylation, protein depolymerization, COPI-coated vesicle-mediated protein transport, and the Ras family proteins. The PPP is essential in producing NADPH, which maintains the cellular redox balance. Moreover, the intermediate products from this pathway contribute to the biosynthesis of many crucial compounds [25]. The respiratory chain is the main pathway to supply energy via ATP production for various biological processes, with the essential components including both NADH dehydrogenase complex and respiratory chain complex I [26,27]. Nucleotide phosphorylation is the upstream pathway in the synthesis of DNA and RNA, serving as a protein production template [28]. Conversely, protein depolymerization is the downstream pathway of protein production. The COPI-coated vesicle-mediate protein transport system from the Golgi to the endoplasmic reticulum is important for the early secretory pathway associated with fungal pathogenicity [29,30]. Furthermore, some members of the Ras family are crucial in the pathogenicity of plant pathogenic fungi [31]. These findings suggest that *Foc* enhances the PPP pathway to provide necessary materials, utilizes the respiratory chain to supply energy for protein production, relies on COPI-related pathways to maintain protein secretion to the host plant, and engages in the biosynthesis of Ras family proteins to enhance virulence during the transition from the saprophytic lifestyle to the parasitic lifestyle. However, the precise roles of enriched acetylated proteins in these processes are not well understood and need to be explored in the future.

Comparative acetylome analyses of two strains of *Foc* with different virulence provided a complete profile of acetylated proteins associated with evolution. The highly virulent strain Ra-4 showed enhanced energy metabolism compared to the mild strain foc-3b, not only during the saprophytic stage but also throughout the parasitic stage. This suggests that robust metabolic activity is essential for achieving high virulence and facilitating adaptive evolution. Moreover, the evolved strain showed much faster responses to the host, exhibiting significantly more intense responses to the host and its defense mechanisms, as well as responses to hypoxia and biofilm formation. These processes are interrelated, as fungal biofilm formation on the surface of hyphae provides a protective barrier, producing hypoxic microenvironments to shield the fungus from host defenses and unfavorable environments [32,33,34].

Notably, the nucleosome and DNA packaging complex associated with gene expression were more active in the evolved strain Ra-4 than the mild strain foc-3b in the parasitic stage, indicating some genes differently expressed between the two strains. This was consistent with our previous comparative transcriptome analyses [9]. In addition, the mild strain foc-3b showed higher hydrogen peroxide activities than the evolved strain Ra-4, suggesting that the evolved strain had adapted to the hydrogen peroxide induced by the host plant defense [35]. Collectively, these comparisons implied that the highly virulent strain Ra-4 evolved faster responses under selective pressures, supported by an ample energy supply. However, many of these acetylated proteins involved in the evolution are unknown. Further functional research on these proteins is necessary to uncover the specific roles of acetylation in the evolution of *Foc*.

While most of the differently acetylated proteins identified do not have well-known functions, some proteins have been extensively studied in several fungal species, such as mitogen-activated protein kinase cascades and cytochrome P450 enzymes [36,37]. For instance, in *F. oxysporum*, FUB1 has been identified as a crucial protein in the synthesis of fusaric acid [38]. However, the specific role of Kac modifications in these proteins, particularly associated with virulence, has not been reported.

It is important to note that the acetylome of parasitic lifestyle in *Foc* was determined at only one-time point in the initial infection stage in this study. Although this time point was crucial for the successful infection of *F. oxysporum* [39], incorporating more time points would provide more valuable information for a comprehensive understanding of the acetylome profile of *Foc* throughout its lifecycle. Moreover, validating the functional significance of Kac modifications in these proteins will help to understand the mechanism of pathogenesis in *Foc* fully.

Cucumber Fusarium wilt caused by *Foc* is a significant threat to the sustainable production of cucumber. While resistant cultivars have been produced through breeding efforts, the virulence of pathogens can evolve, potentially breaking the resistance. This study provides valuable insights into the mechanisms of pathogenesis and virulence evolution in *Foc*, specifically focusing on Kac modifications. These findings can pave the way for novel approaches to effectively manage this pathogen and improve the overall management strategies for combating cucumber Fusarium wilt.

## Figures and Tables

**Table 1:** Eukaryotic Orthologous Group (KOG) functional description of differentially lysine-acetylated proteins in *Fusarium oxysporum* f. sp. *cucumerinum* strains foc-3b (W) and Ra-4 (I) during the saprophytic lifestyle (0 h) in potato dextrose broth cultures (26 °C, 180 rpm, 3 days) or parasitic (6 h) lifestyle 6 h post-inoculation *Cucumis sativus* ‘Zhongnong No. 6′ roots.

Eukaryotic Orthologous Group (KOG) Functional Description	Comparisons			
	W_6h vs. W_0h	I_6h vs. I_0h	I_0h vs. W_0h	I_6h vs. W_6h
[E] Amino acid transport and metabolism	143	139	75	6
[C] Energy production and conversion	132	128	72	10
[O] Posttranslational modification, protein turnover, chaperones	181	174	71	18
[J] Translation, ribosomal structure, and biogenesis	172	172	67	13
[G] Carbohydrate transport and metabolism	95	93	62	11
[S] Function unknown	135	133	57	6
[I] Lipid transport and metabolism	89	81	42	6
[Q] Secondary metabolites biosynthesis, transport, and catabolism	85	80	39	5
[T] Signal transduction mechanisms	106	98	36	1
[U] Intracellular trafficking, secretion, and vesicular transport	127	130	33	9
[K] Transcription	66	67	26	4
[B] Chromatin structure and dynamics	40	40	26	5
[A] RNA processing and modification	78	81	26	2
[H] Coenzyme transport and metabolism	52	50	23	4
[F] Nucleotide transport and metabolism	47	46	21	3
[P] Inorganic ion transport and metabolism	33	31	19	5
[Z] Cytoskeleton	50	49	16	1
[D] Cell cycle control, cell division, chromosome partitioning	38	40	13	2
[M] Cell wall/membrane/envelope biogenesis	17	18	9	2
[L] Replication, recombination, and repair	34	34	7	1
[Y] Nuclear structure	16	15	5	2
[V] Defense mechanisms	7	8	5	2
[W] Extracellular structures	2	2	-	-

**Table 2:** Subcellular location of differentially lysine-acetylated proteins in *Fusarium oxysporum* f. sp. *cucumerinum* strains foc-3b (W) and Ra-4 (I) during the saprophytic lifestyle (0 h, or before inoculation) in potato dextrose broth cultures (26 °C, 180 rpm, 3 days) or parasitic (6 h) lifestyle 6 h post-inoculation *Cucumis sativus* ‘Zhongnong No. 6′ roots.

Subcellular Localization	Comparisons			
	W_6h vs. W_0h	I_6h vs. I_0h	I_0h vs. W_0h	I_6h vs. W_6h
Cytoplasm	642	627	328	64
Nucleus	568	558	217	31
Mitochondria	420	409	170	17
Extracellular	144	140	87	8
Plasma membrane	138	139	65	6
Cytoplasm, nucleus	113	111	51	5
Cytoskeleton	55	51	-	-
Other	17	15	21	2

## Data Availability

All data are included in the main text and Supplementary Materials online.

## Supplementary Materials

The following supporting information can be downloaded at: https://www.mdpi.com/article/10.3390/jof9090920/s1. Table S1: Lysine-acetylated modification sites identified in acetylome of *Fusarium oxysporum* f. sp. *cucumerinum* strains foc-3b and Ra-4. Table S2: Motifs and conservation of lysine-acetylated sites identified in the acetylome of *Fusarium oxysporum* f. sp. *cucumerinum* strains foc-3b and Ra-4. Table S3: Differentially lysine-acetylated proteins and sites identified in *Fusarium oxysporum* f. sp. *cucumerinum* in different combinations. Table S4: *Fusarium oxysporum* f. sp. *cucumerinum* Gene Ontology (GO) functional description of differentially lysine-acetylated proteins in different comparisons. Table S5: *Fusarium oxysporum* f. sp. *cucumerinum* subcellular location of differentially lysine-acetylated proteins in the comparisons. Table S6: *Fusarium oxysporum* f. sp. *cucumerinum* Eukaryotic Orthologous Group (KOG) functional description of differentially lysine-acetylated proteins in different comparisons. Table S7: *Fusarium oxysporum* f. sp. *cucumerinum* Gene Ontology (GO) enrichment of differentially lysine-acetylated proteins in different comparisons. Table S8: *Fusarium oxysporum* f. sp. *cucumerinum* Kyoto Encyclopedia of Genes and Genomes (KEGG) pathway enrichment of differentially lysine-acetylated proteins in different comparisons. Table S9: *Fusarium oxysporum* f. sp. *cucumerinum* protein domain enrichment of differentially lysine-acetylated proteins in different comparisons. Table S10: *Fusarium oxysporum* f. sp. *cucumerinum* closest interaction relationships of differentially lysine-acetylated proteins in different comparisons.
